# Higher blood high density lipoprotein and apolipoprotein A1 levels are associated with reduced risk of developing amyotrophic lateral sclerosis

**DOI:** 10.1136/jnnp-2021-327133

**Published:** 2021-09-13

**Authors:** Alexander G Thompson, Kevin Talbot, Martin R Turner

**Affiliations:** Nuffield Department of Clinical Neurosciences, University of Oxford, Oxford, UK

**Keywords:** epidemiology, cholesterol

## Abstract

**Background:**

Premorbid body mass index, physical activity, diabetes and cardiovascular disease have been associated with an altered risk of developing amyotrophic lateral sclerosis (ALS). There is evidence of shared genetic risk between ALS and lipid metabolism. A very large prospective longitudinal population cohort permits the study of a range of metabolic parameters and the risk of subsequent diagnosis of ALS.

**Methods:**

The risk of subsequent ALS diagnosis in those enrolled prospectively to the UK Biobank (n=502 409) was examined in relation to baseline levels of blood high and low density lipoprotein (HDL, LDL), total cholesterol, total cholesterol:HDL ratio, apolipoproteins A1 and B (apoA1, apoB), triglycerides, glycated haemoglobin A1c (HbA1c) and creatinine, plus self-reported exercise and body mass index.

**Results:**

Controlling for age and sex, higher HDL (HR 0.84, 95% CI 0.73 to 0.96, p=0.010) and apoA1 (HR 0.83, 95% CI 0.72 to 0.94, p=0.005) were associated with a reduced risk of ALS. Higher total cholesterol:HDL was associated with an increased risk of ALS (HR 1.17, 95% CI 1.05 to 1.31, p=0.006). In models incorporating multiple metabolic markers, higher LDL or apoB was associated with an increased risk of ALS, in addition to a lower risk with higher HDL or apoA. Coronary artery disease, cerebrovascular disease and increasing age were also associated with an increased risk of ALS.

**Conclusions:**

The association of HDL, apoA1 and LDL levels with risk of ALS contributes to an increasing body of evidence that the premorbid metabolic landscape may play a role in pathogenesis. Understanding the molecular basis for these changes will inform presymptomatic biomarker development and therapeutic targeting.

## Introduction

Beyond the few monogenetic variants that account for less than 15% of cases, the precise factors underpinning the development of the neurodegenerative disorder amyotrophic lateral sclerosis (ALS) remain unclear.^1 2^ ALS has a consistent incidence of ~2/100 000/year across Europe^3^ and involves a relatively selective degeneration of motor system function, extending to wider cerebral networks in a clinicopathological spectrum with frontotemporal dementia.^4^

Numerous epidemiological studies have considered the role of metabolic factors in the development of ALS. Relative cardiovascular health and lower premorbid body mass index (BMI) have been associated with an increased risk of developing ALS.^5–8^ Physical activity has been inconsistently associated with an increased risk of developing ALS, with a suggestion that strenuous exercise is more specific and may be causally related to susceptibility to ALS.^9–11^ Diabetes appears to modulate the risk of developing ALS in a relationship that varies with age.^12–14^ Higher levels of low density lipoprotein cholesterol (LDL) and apolipoprotein B:A1 have been associated with an increased risk of subsequent ALS, with temporal changes in the lipid biomarker profile observed in the decade prior to diagnosis.^15^ Mendelian randomisation studies have also provided evidence of a causal link for some lipid biomarker levels and ALS.^16 17^

Much of the epidemiological research in ALS has been based on case–control studies, which carry inherent risks of referral, selection and recall bias, issues which can in part be circumvented by prospective cohort studies.^18^ This study sought to examine the relationship between previously highlighted metabolic factors, including blood markers of lipid and carbohydrate metabolism, physical exercise and BMI, with the risk of subsequent development of ALS using data from a very large prospective longitudinal population cohort.

## Methods

### Participants and consent

The UK Biobank is a prospective cohort study of over 500 000 people aged between 39 and 72 years (www.ukbiobank.ac.uk). All people within the specified age range registered with the National Health Service and living within approximately 25 miles of one of the 22 assessment centres distributed around the UK were invited to take part.^19^ Participants underwent initial assessment between March 2006 and October 2010 and were followed for a median of 11.9 years (11.1–12.6 years; table 1). Ethical approval was granted by the Health Research Authority (North West – Haydock Research Ethics Committee reference 16/NW/0274). All participants provided informed electronic consent.

**Table 1:** Cohort baseline demographic and metabolic data

	Entire cohort	ALS	ALS >5 years from enrolment	P value
n	502 409	343	192	–
Age, median (IQR)	58 (50–63)	62 (57–66)	62 (56.75–66)	<0.001†
Age at ALS diagnosis, median (IQR) (years)	–	67.36 (61.71–70.95)	69.17 (63.33–72.35)	–
Latency from enrolment to diagnosis, median (IQR) (days)	–	1986 (1203.5–2444)	2390 (2153–2654)	–
Latency from diagnosis to death, median(95% CI) (days)	–	445 (401 to 542)	508 (401 to 691)	–
% ALS diagnoses from death certificate (n)	–	10 (35)	14 (26)	–
Female participants (%)	273 348 (54.4)	149 (43.4)	84 (43.8)	<0.001*
Follow-up, median (IQR) (days)	4334 (4072–4593)	2491 (1705–3452)	2949 (2499–3882)	–
Total cholesterol, median (IQR) (mmol/L)	5.65 (4.91–6.42)	5.64 (4.85–6.44)	5.81 (4.96–6.53)	0.760†
LDL cholesterol, median (IQR) (mmol/L)	3.52 (2.94–4.12)	3.54 (2.94–4.08)	3.65 (3–4.12)	0.860†
HDL cholesterol, median (IQR) (mmol/L)	1.40 (1.17–1.67)	1.30 (1.1–1.58)	1.29 (1.12–1.55)	<0.001†
Total cholesterol:HDL ratio, median (IQR)	3.97 (3.3–4.81)	4.09 (3.43–5.05)	4.19 (3.53–5.05)	0.007†
Apolipoprotein A, median (IQR) (g/L)	1.51 (1.35–1.7)	1.46 (1.29–1.65)	1.46 (1.3–1.63)	0.002†
Apolipoprotein B, median (IQR) (g/L)	1.02 (0.86–1.18)	1.03 (0.84–1.18)	1.05 (0.89–1.2)	0.902†
Glycated haemoglobin HbA1c, median (IQR) (mmol/mol)	35.2 (32.8–37.9)	35.7 (33.6–38.5)	35.5 (33.4–37.77)	0.022†
Triglycerides, median (IQR) (mmol/L)	1.48 (1.05–2.15)	1.67 (1.18–2.27)	1.68 (1.22–2.23)	<0.001†
Serum creatinine, median (IQR) (µmol/L)	70.4 (61.4–80.9)	71.4 (62.6–81.88)	72.4 (63.38–81.58)	0.106
Excess MET, median (IQR) (hours/week)	23.14 (10.64–46.15)	20.38 (8.45–45.53)	21.46 (10.11–46.25)	0.099†
BMI, median (IQR) (kg/m^2^)	26.74 (24.14–29.91)	27.19 (24.64–30.01)	27.01 (24.78–29.66)	0.111†

Median latency from ALS to diagnosis to death calculated from the Kaplan–Meier survival curve, excluding those in whom the diagnosis is based only on death certificate.

P values indicated for all ALS vs all non-ALS.

*Fisher’s exact test.

†Mann–Whitney U test.

ALS, amyotrophic lateral sclerosis; BMI, body mass index; HbA1c, glycated haemoglobin A1c; HDL, high density lipoprotein; LDL, low density lipoprotein; MET, metabolic equivalent task.

Participants provided demographic and health information alongside donated blood for biochemical analysis performed by the UK Biobank according to standard protocols (https://biobank.ctsu.ox.ac.uk/), which included measurement of blood total cholesterol, high density lipoprotein cholesterol (HDL), LDL, triglycerides, apolipoprotein A1 (apoA1), apolipoprotein B (apoB), HbA1c and creatinine. Excess metabolic equivalent task (MET) hours were calculated based on self-reported walking, moderate and vigorous activity per week.^20^ Participants reported how many days per week they undertook each category of exercise for more than 10 min. The reported duration of activity on a typical day was then multiplied by the number of reported days per week. Excess MET was calculated by subtracting one unit per hour from the total hourly MET values given for each category according to the International Physical Activity Questionnaire (walking 2.3, moderate activity 3.0 and vigorous activity 7.0 excess MET), representing the energy expenditure in excess of that of an inactive person.^21^ A diagnosis of ALS made at some point following the initial sampling was obtained by the UK Biobank using inpatient health records using Hospital Episode Statistics – Admitted Patient Care (HES APC, England), Scottish Morbidity Records (SMR01) and Patient Episode Data for Wales (PEDW), and death certificate linkage (as underlying cause or other position on death register records), using ICD10 code G12.2 or ICD9 code 335.2 (motor neuron disease). Cardiovascular and cerebrovascular disease were modelled as categorical variables using record linkage data indicating a diagnosis of ischaemic heart disease (ICD-10 I20–25) or cerebrovascular disease (ICD-10 I60–69) from hospital or mortality data. Smoking was modelled as a continuous variable as the number of reported daily cigarette packs smoked multiplied by the number of years of smoking.

### Statistical analysis

#### Cohort study

Statistical analysis was performed in R. Only incident ALS cases were included in the analysis—that is, participants diagnosed with ALS following sampling and not reporting a diagnosis of ALS at their baseline study visit, or in whom a diagnosis was identified by medical linkage dated prior to sampling. The primary analysis included data for all incident ALS cases who fulfilled these criteria. Detectable markers of neuronal loss—for example, a rise in neurofilaments and chitinase proteins—have been shown to occur within at least 1 year of the onset of symptoms in carriers of pathogenic genetic variants.^22 23^ With the aim of targeting metabolic changes occurring in the years before this phase of neurodegeneration, secondary analysis was therefore performed using only data from participants linked to an ALS diagnosis more than 5 years after their baseline study visit.

Time-to-event analysis for diagnosis of ALS was performed using Cox proportional hazards modelling from study enrolment. Data are presented as hazard ratios (HR) and 95% CI for a 1 SD rise in levels (variables were mean centred and scaled by SD). LDL, HDL, total cholesterol, total cholesterol:HDL ratio, triglycerides and apolipoproteins A1 and B were selected as well-established lipid cardiovascular risk biomarkers, some of which have been found to be associated with ALS risk previously.^15 24^ In addition, we incorporated variables measuring physical activity (excess MET) and glycaemic control in the form of HbA1c, since these have been associated with ALS risk.^9–15^ We included smoking, cardiovascular disease, cerebrovascular disease and statin use in order to control for confounding from these variables, since they have at times been associated with both metabolic biomarkers and risk of ALS.^25–31^

Models were constructed controlling for age at initial visit and sex. Combined models were also constructed, incorporating demographic variables, cardiovascular and cerebrovascular disease, smoking and statin use along with excess MET and blood biomarkers for all incident ALS cases and, separately, those diagnosed with ALS more than 5 years from enrolment. Separate models were constructed incorporating apoA1 and apoB, and HDL and LDL due to the high degree of correlation of apoA1 with HDL (Spearman’s ρ=0.92, p<0.001) and apoB with LDL (ρ=0.96, p<0.001); total cholesterol was excluded due to the high degree of correlation with LDL (ρ=0.95, p<0.001) and apoB (ρ=0.88, p<0.001). Censoring was performed at the last date of acquisition of hospital and mortality data (31 December 2020). Complete case analysis was performed. The proportional hazards assumption was assessed by visual inspection of Schoenfeld residual plots (see online supplemental figure 1). We also performed Cox modelling stratified by age, which did not significantly influence the results. To allow for multiple comparisons, false discovery rate (FDR)-adjusted p values are also provided.

10.1136/jnnp-2021-327133.supp1 Supplementary data

#### Nested case–control study

In order to examine the temporal relationship of HDL, LDL, total cholesterol:HDL ratio, apoA1 and apoB with onset of ALS, nested case–control analysis was performed. Each participant going on to develop ALS was matched with 20 participants not developing ALS using incidence density matching, participants being matched by age of attending assessment centre in years, month of enrolment ±60 days and sex. The group level relationship between biomarker level and time to ALS diagnosis was examined using linear regression of log-transformed analyte levels, incorporating an interaction between ALS status (ie, going on to develop ALS or not going on to develop ALS) and time.

## Results

After excluding participants reporting a personal medical history of ALS at their initial study visit (n=77), data from 502 409 participants were analysed. Baseline data are shown in table 1. A total of 343 participants obtained a diagnosis of ALS during follow-up, giving a crude incidence of 5.85 per 100 000 per year (95% CI 5.25 to 6.51).

Cox proportional hazard models were constructed examining the association of individual metabolic markers with incident ALS controlling for age at enrolment and sex. The results are summarised in table 2. Hazard ratios (HRs) are indicated for a 1 SD rise in biomarker or metabolic parameter level. Incorporating all incident ALS cases, higher HDL (HR 0.84, 95% CI 0.73 to 0.96, p=0.010, adjusted p=0.035) and higher apoA1 (HR 0.83, 95% CI 0.72 to 0.94, p=0.005, adjusted p=0.031) were associated with a reduced risk of subsequent diagnosis of ALS. Higher total cholesterol:HDL (HR 1.17, 95% CI 1.05 to 1.31, p=0.006, adjusted p=0.031) was associated with an increased risk of ALS.

**Table 2:** Cox proportional hazards modelling for individual variables controlling for age at first study visit and sex

	All data					Excluding ALS within 5 years				
	n	Cases	HR (95% CI)	P value	Adjusted p value	n	Cases	HR (95% CI)	P value	Adjusted p value
Total cholesterol	469 500	326	1.00 (0.90 to 1.12)	0.960	0.960	469 354	180	1.07 (0.93 to 1.24)	0.361	0.512
Total cholesterol:HDL ratio	429 710	294	**1.17 (1.05 to 1.31**)	**0.006**	**0.031**	429 578	162	**1.19 (1.03 to 1.39**)	**0.022**	0.123
LDL cholesterol	468 618	325	1.01 (0.91 to 1.12)	0.854	0.960	468 472	179	1.11 (0.96 to 1.28)	0.154	0.356
HDL cholesterol	429 789	294	**0.84 (0.73 to 0.96**)	**0.010**	**0.035**	429 657	162	**0.81 (0.67 to 0.97**)	**0.022**	0.123
Triglycerides	469 126	326	1.08 (0.97 to 1.20)	0.148	0.325	468 980	180	1.04 (0.90 to 1.20)	0.617	0.754
Apolipoprotein A1	427 427	291	**0.83 (0.72 to 0.94**)	**0.005**	**0.031**	427 297	161	**0.83 (0.70 to 0.99**)	**0.043**	0.157
Apolipoprotein B	467 121	324	1.01 (0.90 to 1.12)	0.916	0.960	466 976	179	1.11 (0.96 to 1.28)	0.162	0.356
HbA1c	466 412	323	0.98 (0.88 to 1.10)	0.770	0.960	466 267	178	0.93 (0.78 to 1.10)	0.373	0.512
Excess MET	402 300	275	0.93 (0.82 to 1.05)	0.259	0.475	402 179	154	0.99 (0.84 to 1.16)	0.882	0.882
BMI	499 314	336	1.04 (0.82 to 1.17)	0.444	0.698	499 166	188	1.08 (0.93 to 1.25)	0.302	0.512
Creatinine	469 268	326	0.86 (0.74 to 1.02)	0.076	0.210	469 122	180	0.96 (0.79 to 1.17)	0.696	0.765

Hazard ratios indicated for a 1 SD increase in variable level.

ALS, amyotrophic lateral sclerosis; BMI, body mass index; HbA1c, glycated haemoglobin A1c; HDL, high density lipoprotein; HR, hazard ratio; LDL, low density lipoprotein; MET, metabolic equivalent task.

Models excluding participants diagnosed within 5 years of their initial visit were largely consistent with models incorporating all participants in magnitude and direction of associations, although lipid associations were not significant following correction for multiple comparisons. Higher HDL (HR 0.81, 95% CI 0.67 to 0.97, p=0.022, adjusted p=0.123) and higher apoA1 (HR 0.83, 95% CI 0.70 to 0.99, p=0.043, adjusted p=0.157) were associated with a reduced risk of ALS (table 2). Higher total cholesterol:HDL (HR 1.19, 95% CI 1.03 to 1.39, p=0.022, adjusted p=0.123) was associated with an increased risk of ALS.

Combined models were constructed incorporating HDL and LDL and, separately, apoA1 and apoB (given the high degree of correlation of HDL and LDL with apoA1 and apoB, respectively), with HbA1c, triglycerides, excess MET, BMI, serum creatinine, sex and age. Given the association of vascular diseases with both ALS risk and blood lipid levels,^30–32^ coronary artery and cerebrovascular disease were added as covariates, along with smoking which has been associated with HDL cholesterol levels and risk of ALS,^25–29^ and statin use, which are used in the treatment of hypercholesterolaemia and have been associated with both higher and lower risk of ALS.^33 34^ Total cholesterol was excluded due to a high degree of correlation with LDL. Higher HDL (HR 0.78, 95% CI 0.64 to 0.96, p=0.017, adjusted p=0.054) and ApoA1 (HR 0.82, 95% CI 0.68 to 0.97, p=0.024, adjusted p=0.088) levels were associated with a lower risk of ALS. Excluding those diagnosed within 5 years of their first visit, higher HDL (HR 0.66, 95% CI 0.49 to 0.88, p=0.005, adjusted p=0.020) or apoA1 (HR 0.77, 95% CI 0.61 to 0.98, p=0.034, adjusted p=0.110) levels were associated with a reduced risk of ALS. Higher LDL (HR 1.35, 95% CI 1.09 to 1.68, p=0.007, adjusted p=0.021) or apoB (HR 1.23, 95% CI 1.00 to 1.25, p=0.050, adjusted p=0.129) were associated with an increased risk of ALS. Smoking was not significant in combined models whereas age, cerebrovascular disease and coronary artery disease were associated with higher ALS risk in all models (table 3).

**Table 3:** Combined Cox proportional hazards modelling, controlling for age at first study visit and sex

	All participants						Excluding ALS within 5 years					
	Model 1			Model 2			Model 3			Model 4		
	HR (95% CI)	P value	FDR p value	HR (95% CI)	P value	FDR p value	HR (95% CI)	P value	FDR p value	HR (95% CI)	P value	FDR p value
LDL cholesterol	1.12 (0.95 to 1.32)	0.192	0.356	–	–	–	**1.35 (1.09 to 1.68**)	**0.007**	**0.021**	–	–	–
HDL cholesterol	**0.78 (0.64 to 0.96**)	**0.017**	0.054	–	–	–	**0.66 (0.49 to 0.88**)	**0.005**	**0.020**	–	–	–
Apolipoprotein B	–	–	–	1.06 (0.90 to 1.24)	0.507	0.638	–	–	–	**1.23 (1.00 to 1.52**)	**0.050**	0.129
Apolipoprotein A1	–	–	–	**0.82 (0.68 to 0.97**)	**0.024**	0.088	–	–	–	**0.77 (0.61 to 0.98**)	**0.034**	0.110
Triglycerides	1.01 (0.87 to 1.19)	0.865	0.865	1.04 (0.89 to 1.21)	0.617	0.638	0.86 (0.68 to 1.08)	0.195	0.329	0.94 (0.75 to 1.17)	0.571	0.675
HbA1c	0.97 (0.83 to 1.12)	0.649	0.767	0.96 (0.82 to 1.12)	0.621	0.638	0.87 (0.69 to 1.10)	0.253	0.365	0.86 (0.68 to 1.09)	0.219	0.356
Excess MET	1.00 (0.99 to 1.00)	0.253	0.411	1.00 (0.99 to 1.00)	0.28	0.616	1.00 (0.99 to 1.00)	0.771	0.835	1.00 (0.99 to 1.00)	0.749	0.811
BMI	1.01 (0.97 to 1.04)	0.732	0.792	1.01 (0.97 to 1.04)	0.635	0.638	1.03 (0.99 to 1.08)	0.187	0.329	1.03 (0.99 to 1.08)	0.155	0.337
Creatinine	1.00 (0.99 to 1.01)	0.408	0.589	1.00 (0.99 to 1.01)	0.418	0.638	1.00 (0.99 to 1.01)	0.849	0.849	1.00 (0.99 to 1.01)	0.917	0.917
Smoking pack years	1.00 (0.99 to 1.01)	0.629	0.767	1.00 (0.99 to 1.01)	0.638	0.638	1.01 (1.00 to 1.02)	0.202	0.329	1.01 (1.00 to 1.02)	0.211	0.356
Cardiovascular disease	**1.66 (1.10 to 2.49**)	**0.015**	0.054	**1.69 (1.12 to 2.54**)	**0.012**	0.066	**1.96 (1.16 to 3.30**)	**0.012**	**0.030**	**1.95 (1.16 to 3.29**)	**0.012**	0.052
Cerebrovascular disease	**3.25 (1.96 to 5.38**)	**<0.001**	**<0.001**	**3.29 (1.99 to 5.45**)	**<0.001**	**<0.001**	**3.94 (2.11 to 7.39**)	**<0.001**	**<0.001**	**3.92 (2.09 to 7.34**)	**<0.001**	**<0.001**
Statin use	0.68 (0.43 to 1.08)	0.104	0.226	0.64 (0.41 to 1.00)	0.052	0.143	0.77 (0.42 to 1.42)	0.404	0.477	0.71 (0.39 to 1.29)	0.261	0.361

Four separate models were constructed for HDL and LDL cholesterol, and apoA1 and ApoA2, including and excluding participants going on to develop ALS within 5 years of sampling. Hazard ratios indicated for a 1 SD increase in variable level.

ALS, amyotrophic lateral sclerosis; BMI, body mass index; FDR, false discovery rate-adjusted p value; HbA1c, glycated haemoglobin A1c; HDL, high density lipoprotein; LDL, low density lipoprotein; MET, metabolic equivalent task.

**Figure 1:**
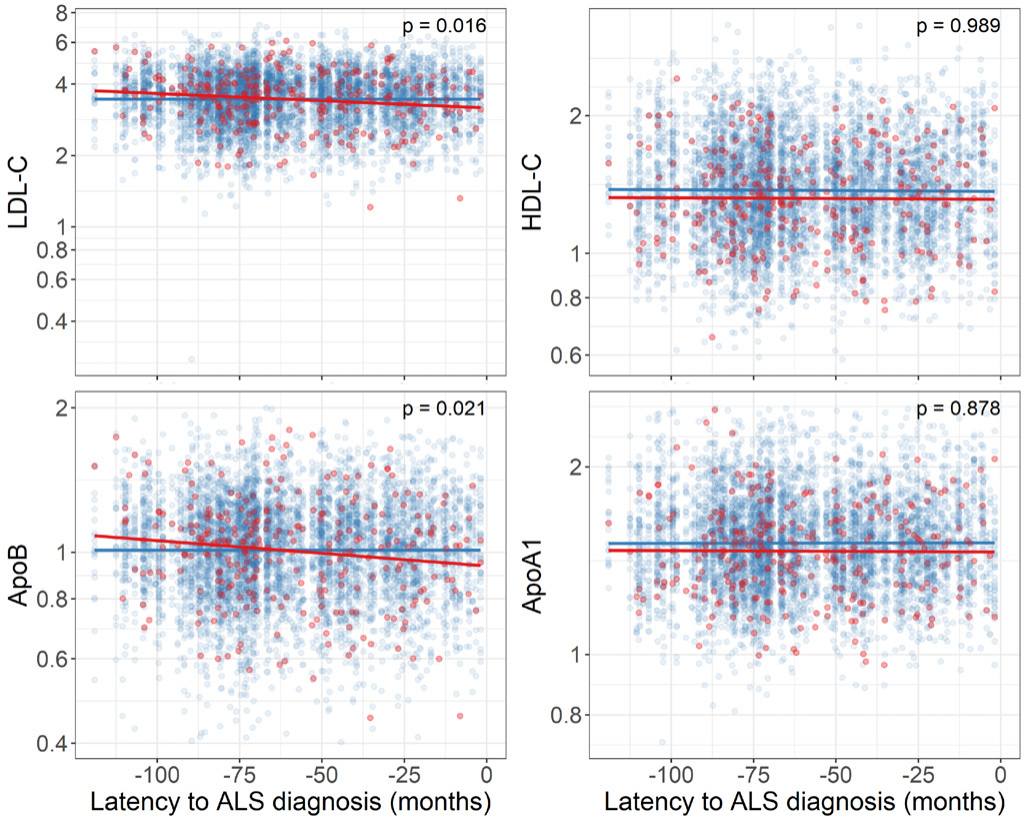
Temporal differences in LDL and HDL cholesterol, ApoB and apoA1 and diagnosis of ALS. Participants going on to develop ALS (red) were each matched by age of sampling in years, date of sampling ±60 days and sex to 20 participants not going on to develop ALS (blue). Lines indicate linear model fit for ALS (red) and non-ALS (blue) participants. P values indicate interaction between ALS status and time, indicating difference in temporal trajectory of biomarkers over time. ApoA1, apolipoprotein A1; ApoB, apolipoprotein B; ALS, amyotrophic lateral sclerosis; HDL-C, high density lipoprotein cholesterol; LDL-C, low density lipoprotein cholesterol.

Nested case–control analysis was used to examine the temporal relationship between ALS diagnosis and lipid biomarker levels for HDL and LDL cholesterol, apoA1 and apoB, matching each participant going on to develop ALS with 20 other participants by age at sampling, month of sampling and sex. Linear models identified significant differences in LDL and apoB slope in participants going on to develop ALS compared with healthy controls, with a downward slope observed in patients with ALS (time–ALS status interaction: LDL p=0.016, apoB p=0.021, figure 1). No significant interaction was observed for HDL or apoA1.

## Discussion

This study used data from a large, prospectively acquired, longitudinal cohort study to examine the relationship between levels of available lipid and broader metabolic parameters and risk of a subsequent diagnosis of ALS. The key finding is that higher levels of HDL and apoA1, with correspondingly lower total cholesterol:HDL at study enrolment, were associated with a reduced risk of ALS diagnosis during follow-up, independent of age and sex. Excluding participants with a short latency from initial sampling to diagnosis, with the aim of restricting analysis prior to the onset of rapid neurodegeneration in ALS, had no material effect on the results. In combined models, HDL and apoA1 remained independent of other factors, and lower LDL and apoB levels were also associated with a decreased risk of ALS. The persistence of these findings in models controlling for statin use, smoking and vascular disease indicates that the association of lipid levels and ALS is not attributable to a confounding association between lipids, ALS and these factors. Nested case–control analysis also identified temporal variation in LDL and apoB levels, decreasing towards diagnosis in those going on to develop ALS, but stable levels of HDL and apoA1.

The results of this study are usefully compared with a population-based analysis of longitudinal primary care data. The Apolipoprotein-related MOrtality RISk study (AMORIS) identified an association between higher LDL:HDL and increased risk of developing ALS.^15^ The increased risk was driven primarily by higher levels of LDL and apoB, whereas in this study the associations mainly reflect relatively lower levels of HDL (or apoA1) measured at enrolment in those subsequently developing ALS, although higher LDL (or apoB) was strongly associated with an increased risk of ALS in a subset of multivariate models. The temporal changes observed in the nested case–control analysis presented here, suggesting decreasing levels approaching diagnosis, mirror those observed in AMORIS participants, which suggests that the differences between studies with respect to LDL and apoB might well relate to the timing of sampling in relation to ALS diagnosis.

A nested case–control analysis combining five cohort studies reported *higher* levels of HDL as a risk factor for ALS,^35^ in contrast to the findings described here. This could also be explained in relation to temporal changes in lipid levels described in the AMORIS study, in which HDL levels are lower in ALS patients 5–15 years prior to diagnosis, although the data presented here indicate stable HDL levels over the years before ALS diagnosis. There might also be differences arising from the timing of ascertainment of ALS diagnosis (the majority being identified from death certification only compared with just 10% of cases in this study), so that a higher proportion of participants were in the early stages of ALS.

The role of lipid metabolism in ALS is complex. In addition to epidemiological studies implicating cholesterol and apolipoprotein biology in the years prior to ALS diagnosis, a body of evidence suggests contrasting effects during the symptomatic phase of the disease. Unlike the association of higher LDL and apoB and lower HDL and apoA1 with a higher risk of ALS in the presymptomatic literature, higher levels of total and LDL cholesterol as well as triglycerides (and in some cases lower HDL) are associated with less rapid disability progression and better respiratory function and survival.^36–39^ Whether this is a protective effect of lipids or a proxy for other factors portending poor prognosis such as lower BMI is not resolved.^6^ However, this highlights the need to distinguish the symptomatic and presymptomatic phases of ALS as much as possible.

Observational studies cannot disentangle the causal direction of the association between HDL and the subsequent development of ALS. Evidence from multiple sources supports shared genetic risk between ALS and lipid profiles.^16 17 40–42^ This has not been convincingly established for HDL though, with one Mendelian randomisation study not finding a causal relationship^16^ and one meta-analysis of genome-wide association study data indicating shared polygenic risk.^42^

The mechanisms by which HDL and ApoA1 might confer increased risk of ALS has not been studied, although they are implicated in multiple pathways of relevance to ALS. ApoA1 forms the main lipoprotein constituent of HDL particles and is essential for reverse cholesterol transport, the process by which cholesterol is removed from peripheral tissues and transported to the liver.^43^ ApoB is the major constituent of LDL particles as well as chylomicrons, very low density lipoprotein and intermediate density lipoprotein particles; thus there is typically a high degree of correlation between HDL and apoA1 levels, and between LDL and apoB levels in serum.^43^ Both HDL and apoA1 have anti-inflammatory effects, reducing monocyte migration and dendritic cell function.^44^ HDL and apoA1 are also antioxidant and preserve mitochondrial function in models of ischaemic heart disease.^45^ Increased cerebrospinal fluid HDL and apoA1 has also been observed following spinal cord injury, and exogenous HDL enhances neuronal growth via the ERK pathway.^46^

Beyond the lipid data, we found significant associations of cardiovascular diseases—both cerebrovascular disease and coronary artery disease—with ALS in multivariate models independent of lipid levels, statin use, HbA1c and smoking. Cerebrovascular disease has been associated with a higher risk of ALS, in keeping with our study.^32^ The nature of the relationship between coronary artery disease and risk of ALS is less clear, with associations between higher and lower levels of coronary artery disease and ALS identified in previous studies.^7 29 30^

Potential reasons for this discrepancy are that, in this study, we have considered coronary artery disease before and after diagnosis of ALS (our aim being to control for cardiovascular diseases confounding associations between metabolic markers and ALS rather than to specifically address the risk of ALS in the presence of coronary artery disease), due to selection bias or to the method of ascertaining ALS or coronary artery disease cases based on hospital inpatient records in this or previous studies, which could enrich for coincident diagnoses through collider bias.^7 29 47^

Statin use has previously been implicated in increased ALS risk through pharmaceutical surveillance,^33^ although this has largely been refuted by recent unbiased population-based studies which failed to identify any association between statin use and risk of ALS.^34 48^ This study incorporated statin use as a covariate primarily to exclude it as a confounder for lipid biomarkers and ALS, but its findings are in keeping with the recent literature. Similarly, there is substantial evidence that smoking increases the risk of ALS, both through traditional epidemiological studies and Mendelian randomisation approaches exploring causality (although there is not complete agreement between studies).^25–27 49 50^ This study has not sought to elucidate this question further, but indicates that the associations between lipid biomarkers and ALS identified here are independent of lifetime tobacco exposure as measured in pack-years.

Although these data do not lend support to the previously identified association between exercise and risk of ALS, it should be noted that our use of excess MET as a continuous variable encompassing any activity from walking to vigorous exercise does not address a specific role for frequent vigorous exercise and ALS risk, as implicated in recent Mendelian randomisation analysis of causality.^11^

It is likely that, due to case ascertainment by inpatient record linkage in the UK Biobank, the latency from symptom onset to date of diagnosis will be longer in these data than using outpatient encounters. This is reflected in the older age of onset in ALS cases in the UK Biobank cohort and relatively short latency to death compared with other studies.^51^ Our restricted analysis, including only those ALS cases identified more than 5 years after sampling, mitigates that issue.

Limitations relating to the recruitment of participants in the UK Biobank are also recognised and might contribute to the observed differences in relation to previously published data. Participants in the UK Biobank differ from the general population of the UK in indices of lifestyle, ethnicity, health and wealth^52^; it is recognised that this can lead to biased estimates, which cannot be excluded in this or other epidemiological analyses of lipids in ALS.^47^ This may have contributed to the apparent older age of ALS diagnosis in the UK Biobank when compared with other studies. The selection of participants by age range could exclude older or younger people developing ALS; this proportion would be expected to be small, however, since most people developing ALS do so between the ages of 40 and 70, and since the follow-up in the UK Biobank (median >11 years, table 1) would capture much of the remainder who develop ALS by 80 years.^53^ The incidence of ALS in the UK Biobank is similar to the incidence reported in other countries in Europe and North America, allowing for the age range of participants.^54^

Incorrect identification of ALS cases is still possible within the UK Biobank due to the reliance on record linkage and the use of ICD10 and ICD9 codes that encompass non-ALS motor neuron diseases such as primary lateral sclerosis. Validation studies suggest that the positive predictive value of such methodologies is 70–91%,^55^ and any diluting effect of rare motor neuron disorders is expected to be small. Additional limitations are the lack of data on common genetic causes of ALS, which might influence lipid biomarkers, and the potential for masked confounding effects.

This study adds to a growing literature documenting differences in the premorbid metabolic profile of those who eventually develop ALS. In addition to providing novel insights into pathogenesis, this emphasises the need to consider a broader set of potential presymptomatic ALS biomarkers. Such markers might help to target population screening for ALS and also build confidence in future trials of preventative therapy.

## Data Availability

Data may be obtained from a third party and are not publicly available.

## References

[1] Al-Chalabi A , Calvo A , Chio A , et al . Analysis of amyotrophic lateral sclerosis as a multistep process: a population-based modelling study. Lancet Neurol 2014;13:1108–13. 10.1016/S1474-4422(14)70219-4 25300936 PMC4197338

[2] Talbot K , Feneberg E , Scaber J , et al . Amyotrophic lateral sclerosis: the complex path to precision medicine. J Neurol 2018;265:2454–62. 10.1007/s00415-018-8983-8 30054789 PMC6182683

[3] Chiò A , Logroscino G , Traynor BJ , et al . Global epidemiology of amyotrophic lateral sclerosis: a systematic review of the published literature. Neuroepidemiology 2013;41:118–30. 10.1159/000351153 23860588 PMC4049265

[4] Turner MR , Swash M . The expanding syndrome of amyotrophic lateral sclerosis: a clinical and molecular odyssey. J Neurol Neurosurg Psychiatry 2015;86:667–73. 10.1136/jnnp-2014-308946 25644224 PMC4453495

[5] O'Reilly ÉJ. , Wang H , Weisskopf MG , et al . Premorbid body mass index and risk of amyotrophic lateral sclerosis. Amyotroph Lateral Scler Frontotemporal Degener 2013;14:205–11. 10.3109/21678421.2012.735240 23134505 PMC3615420

[6] Paganoni S , Deng J , Jaffa M , et al . Body mass index, not dyslipidemia, is an independent predictor of survival in amyotrophic lateral sclerosis. Muscle Nerve 2011;44:20–4. 10.1002/mus.22114 21607987 PMC4441750

[7] Turner MR , Wotton C , Talbot K , et al . Cardiovascular fitness as a risk factor for amyotrophic lateral sclerosis: indirect evidence from record linkage study. J Neurol Neurosurg Psychiatry 2012;83:395–8. 10.1136/jnnp-2011-301161 22072701

[8] Sutedja NA , van der Schouw YT , Fischer K , et al . Beneficial vascular risk profile is associated with amyotrophic lateral sclerosis. J Neurol Neurosurg Psychiatry 2011;82:638–42. 10.1136/jnnp.2010.236752 21471184

[9] Huisman MHB , Seelen M , de Jong SW , et al . Lifetime physical activity and the risk of amyotrophic lateral sclerosis. J Neurol Neurosurg Psychiatry 2013;84:976–81. 10.1136/jnnp-2012-304724 23418211

[10] Fang F , Hållmarker U , James S , et al . Amyotrophic lateral sclerosis among cross-country skiers in Sweden. Eur J Epidemiol 2016;31:247–53. 10.1007/s10654-015-0077-7 26220522

[11] Julian TH , Glascow N , Barry ADF , et al . Physical exercise is a risk factor for amyotrophic lateral sclerosis: convergent evidence from mendelian randomisation, transcriptomics and risk genotypes. EBioMedicine 2021;68:103397. 10.1016/j.ebiom.2021.103397 34051439 PMC8170114

[12] Kioumourtzoglou M-A , Rotem RS , Seals RM , et al . Diabetes mellitus, obesity, and diagnosis of amyotrophic lateral sclerosis: a population-based study. JAMA Neurol 2015;72:905–11. 10.1001/jamaneurol.2015.0910 26030836 PMC4975611

[13] Mariosa D , Kamel F , Bellocco R , et al . Association between diabetes and amyotrophic lateral sclerosis in Sweden. Eur J Neurol 2015;22:1436–42. 10.1111/ene.12632 25600257 PMC4506907

[14] Sun Y , Lu C-J , Chen R-C , et al . Risk of amyotrophic lateral sclerosis in patients with diabetes: a nationwide population-based cohort study. J Epidemiol 2015;25:445–51. 10.2188/jea.JE20140176 25947580 PMC4444499

[15] Mariosa D , Hammar N , Malmström H , et al . Blood biomarkers of carbohydrate, lipid, and apolipoprotein metabolisms and risk of amyotrophic lateral sclerosis: a more than 20-year follow-up of the Swedish AMORIS cohort. Ann Neurol 2017;81:718–28. 10.1002/ana.24936 28437840

[16] Zeng P , Zhou X . Causal effects of blood lipids on amyotrophic lateral sclerosis: a Mendelian randomization study. Hum Mol Genet 2019;28:688–97. 10.1093/hmg/ddy384 30445611 PMC6360326

[17] Bandres-Ciga S , Noyce AJ , Hemani G , et al . Shared polygenic risk and causal inferences in amyotrophic lateral sclerosis. Ann Neurol 2019;85:470–81. 10.1002/ana.25431 30723964 PMC6450729

[18] Breslow NE . Statistics in epidemiology: the case-control study. J Am Stat Assoc 1996;91:14–28. 10.1080/01621459.1996.10476660 12155399

[19] Allen N , Sudlow C , Downey P , et al . UK Biobank: current status and what it means for epidemiology. Health Policy Technol 2012;1:123–6. 10.1016/j.hlpt.2012.07.003

[20] Ainsworth BE , Haskell WL , Herrmann SD , et al . 2011 compendium of physical activities: a second update of codes and MET values. Med Sci Sports Exerc 2011;43:1578–81. 10.1249/MSS.0b013e31821ece12 21681120

[21] Bradbury KE , Guo W , Cairns BJ , et al . Association between physical activity and body fat percentage, with adjustment for BMI: a large cross-sectional analysis of UK biobank. BMJ Open 2017;7:e011843. 10.1136/bmjopen-2016-011843 PMC5372047 28341684

[22] Benatar M , Wuu J , Andersen PM , et al . Neurofilament light: a candidate biomarker of presymptomatic amyotrophic lateral sclerosis and phenoconversion. Ann Neurol 2018;84:130–9. 10.1002/ana.25276 30014505 PMC11348288

[23] Gray E , Thompson AG , Wuu J , et al . CSF chitinases before and after symptom onset in amyotrophic lateral sclerosis. Ann Clin Transl Neurol 2020;7:1296–306. 10.1002/acn3.51114 32666680 PMC7448184

[24] Castelli WP , Anderson K , Wilson PW , et al . Lipids and risk of coronary heart disease. study. Ann Epidemiol 1992;2:23–8. 10.1016/1047-2797(92)90033-M 1342260

[25] Opie-Martin S , Jones A , Iacoangeli A , et al . UK case control study of smoking and risk of amyotrophic lateral sclerosis. Amyotroph Lateral Scler Frontotemporal Degener 2020;21:222–7. 10.1080/21678421.2019.1706580 32301340 PMC7261396

[26] Zhan Y , Fang F . Smoking and amyotrophic lateral sclerosis: a Mendelian randomization study. Ann Neurol 2019;85:482–4. 10.1002/ana.25443 30786056

[27] Opie-Martin S , Wootton RE , Budu-Aggrey A , et al . Relationship between smoking and ALS: Mendelian randomisation interrogation of causality. J Neurol Neurosurg Psychiatry 2020;91:1312–5. 10.1136/jnnp-2020-323316 32848012

[28] Gallo V , Bueno-De-Mesquita HB , Vermeulen R , et al . Smoking and risk for amyotrophic lateral sclerosis: analysis of the EPIC cohort. Ann Neurol 2009;65:378–85. 10.1002/ana.21653 19399866

[29] Pereira M , Gromicho M , Henriques A , et al . Cardiovascular comorbidities in amyotrophic lateral sclerosis. J Neurol Sci 2021;421:117292. 10.1016/j.jns.2020.117292 33423011

[30] Kioumourtzoglou M-A , Seals RM , Gredal O , et al . Cardiovascular disease and diagnosis of amyotrophic lateral sclerosis: a population based study. Amyotroph Lateral Scler Frontotemporal Degener 2016;17:548–54. 10.1080/21678421.2016.1208247 27436717 PMC5178102

[31] Wilson PW , Abbott RD , Castelli WP . High density lipoprotein cholesterol and mortality. The Framingham Heart Study. Arteriosclerosis 1988;8:737–41. 10.1161/01.ATV.8.6.737 3196218

[32] Turner MR , Goldacre R , Talbot K , et al . Cerebrovascular injury as a risk factor for amyotrophic lateral sclerosis. J Neurol Neurosurg Psychiatry 2016;87:244–6. 10.1136/jnnp-2015-311157 26260352 PMC4789816

[33] Golomb BA , Verden A , Messner AK , et al . Amyotrophic lateral sclerosis associated with statin use: a disproportionality analysis of the FDA's adverse event reporting system. Drug Saf 2018;41:403–13. 10.1007/s40264-017-0620-4 29427042

[34] Mariosa D , Kamel F , Bellocco R , et al . Antidiabetics, statins and the risk of amyotrophic lateral sclerosis. Eur J Neurol 2020;27:1010–6. 10.1111/ene.14190 32097525 PMC10957794

[35] Bjornevik K , O'Reilly Éilis J , Cortese M , et al . Pre-diagnostic plasma lipid levels and the risk of amyotrophic lateral sclerosis. Amyotroph Lateral Scler Frontotemporal Degener 2021;22:133–43. 10.1080/21678421.2020.1822411 32985910 PMC8004541

[36] Ingre C , Chen L , Zhan Y , et al . Lipids, apolipoproteins, and prognosis of amyotrophic lateral sclerosis. Neurology 2020;94:e1835–44. 10.1212/WNL.0000000000009322 32221024 PMC7274849

[37] Chiò A , Calvo A , Ilardi A , et al . Lower serum lipid levels are related to respiratory impairment in patients with ALS. Neurology 2009;73:1681–5. 10.1212/WNL.0b013e3181c1df1e 19917991

[38] Ikeda K , Hirayama T , Takazawa T , et al . Relationships between disease progression and serum levels of lipid, urate, creatinine and ferritin in Japanese patients with amyotrophic lateral sclerosis: a cross-sectional study. Intern Med 2012;51:1501–8 https://www.ncbi.nlm.nih.gov/pubmed/22728481 10.2169/internalmedicine.51.7465 22728481

[39] Dorst J , Kühnlein P , Hendrich C , et al . Patients with elevated triglyceride and cholesterol serum levels have a prolonged survival in amyotrophic lateral sclerosis. J Neurol 2011;258:613–7. 10.1007/s00415-010-5805-z 21128082

[40] De Aguilar JLG . Lipid biomarkers for amyotrophic lateral sclerosis. Front Neurol 2019;10:248. 10.3389/fneur.2019.00284 31019485 PMC6458258

[41] Nakamura R , Misawa K , Tohnai G , et al . A multi-ethnic meta-analysis identifies novel genes, including ACSL5, associated with amyotrophic lateral sclerosis. Commun Biol 2020;3:526. 10.1038/s42003-020-01251-2 32968195 PMC7511394

[42] Li C , Ou R , Wei Q , et al . Shared genetic links between amyotrophic lateral sclerosis and obesity-related traits: a genome-wide association study. Neurobiol Aging 2021;102:211.e1:211–211.e9. 10.1016/j.neurobiolaging.2021.01.023 33640203

[43] Andrikoula M , McDowell IFW . The contribution of ApoB and ApoA1 measurements to cardiovascular risk assessment. Diabetes Obes Metab 2008;10:271–8. 10.1111/j.1463-1326.2007.00714.x 18333887

[44] Tiniakou I , Drakos E , Sinatkas V , et al . High-density lipoprotein attenuates Th1 and Th17 autoimmune responses by modulating dendritic cell maturation and function. J Immunol 2015;194:4676–87. 10.4049/jimmunol.1402870 25870241 PMC4417411

[45] White CR , Datta G , Giordano S . High-density lipoprotein regulation of mitochondrial function. Adv Exp Med Biol 2017;982:407–29. 10.1007/978-3-319-55330-6_22 28551800 PMC5822681

[46] Sengupta MB , Saha S , Mohanty PK , et al . Increased expression of ApoA1 after neuronal injury may be beneficial for healing. Mol Cell Biochem 2017;424:45–55. 10.1007/s11010-016-2841-8 27734225

[47] Munafò MR , Tilling K , Taylor AE , et al . Collider scope: when selection bias can substantially influence observed associations. Int J Epidemiol 2018;47:226–35. 10.1093/ije/dyx206 29040562 PMC5837306

[48] Sørensen HT , Riis AH , Lash TL , et al . Statin use and risk of amyotrophic lateral sclerosis and other motor neuron disorders. Circ Cardiovasc Qual Outcomes 2010;3:413–7. 10.1161/CIRCOUTCOMES.110.936278 20530788

[49] Westeneng H-J , van Veenhuijzen K , van der Spek RA , et al . Associations between lifestyle and amyotrophic lateral sclerosis stratified by C9orf72 genotype: a longitudinal, population-based, case-control study. Lancet Neurol 2021;20:373–84. 10.1016/S1474-4422(21)00042-9 33894192

[50] Wang H , O'Reilly Éilis J , Weisskopf MG , et al . Smoking and risk of amyotrophic lateral sclerosis: a pooled analysis of 5 prospective cohorts. Arch Neurol 2011;68:207–13. 10.1001/archneurol.2010.367 21320987 PMC3319086

[51] Logroscino G , Traynor BJ , Hardiman O , et al . Incidence of amyotrophic lateral sclerosis in Europe. J Neurol Neurosurg Psychiatry 2010;81:385–90. 10.1136/jnnp.2009.183525 19710046 PMC2850819

[52] Fry A , Littlejohns TJ , Sudlow C , et al . Comparison of sociodemographic and health-related characteristics of UK Biobank participants with those of the general population. Am J Epidemiol 2017;186:1026–34. 10.1093/aje/kwx246 28641372 PMC5860371

[53] Longinetti E , Regodón Wallin A , Samuelsson K , et al . The Swedish Motor Neuron Disease Quality Registry. Amyotroph Lateral Scler Front Degener 2018;19:528–37. 10.1080/21678421.2018.1497065 30296856

[54] Cronin S , Hardiman O , Traynor BJ . Ethnic variation in the incidence of ALS: a systematic review. Neurology 2007;68:1002–7. 10.1212/01.wnl.0000258551.96893.6f 17389304

[55] Horrocks S , Wilkinson T , Schnier C , et al . Accuracy of routinely-collected healthcare data for identifying motor neurone disease cases: a systematic review. PLoS One 2017;12:e0172639. 10.1371/journal.pone.0172639 28245254 PMC5330471

