# Supplementary figures and images for: Higher blood high density lipoprotein and apolipoprotein A1 levels are associated with reduced risk of developing amyotrophic lateral sclerosis
Source: J Neurol Neurosurg Psychiatry. 2021 Sep 13;93(1):75–81. doi: 10.1136/jnnp-2021-327133 (PMC8685635; doi:10.1136/jnnp-2021-327133)

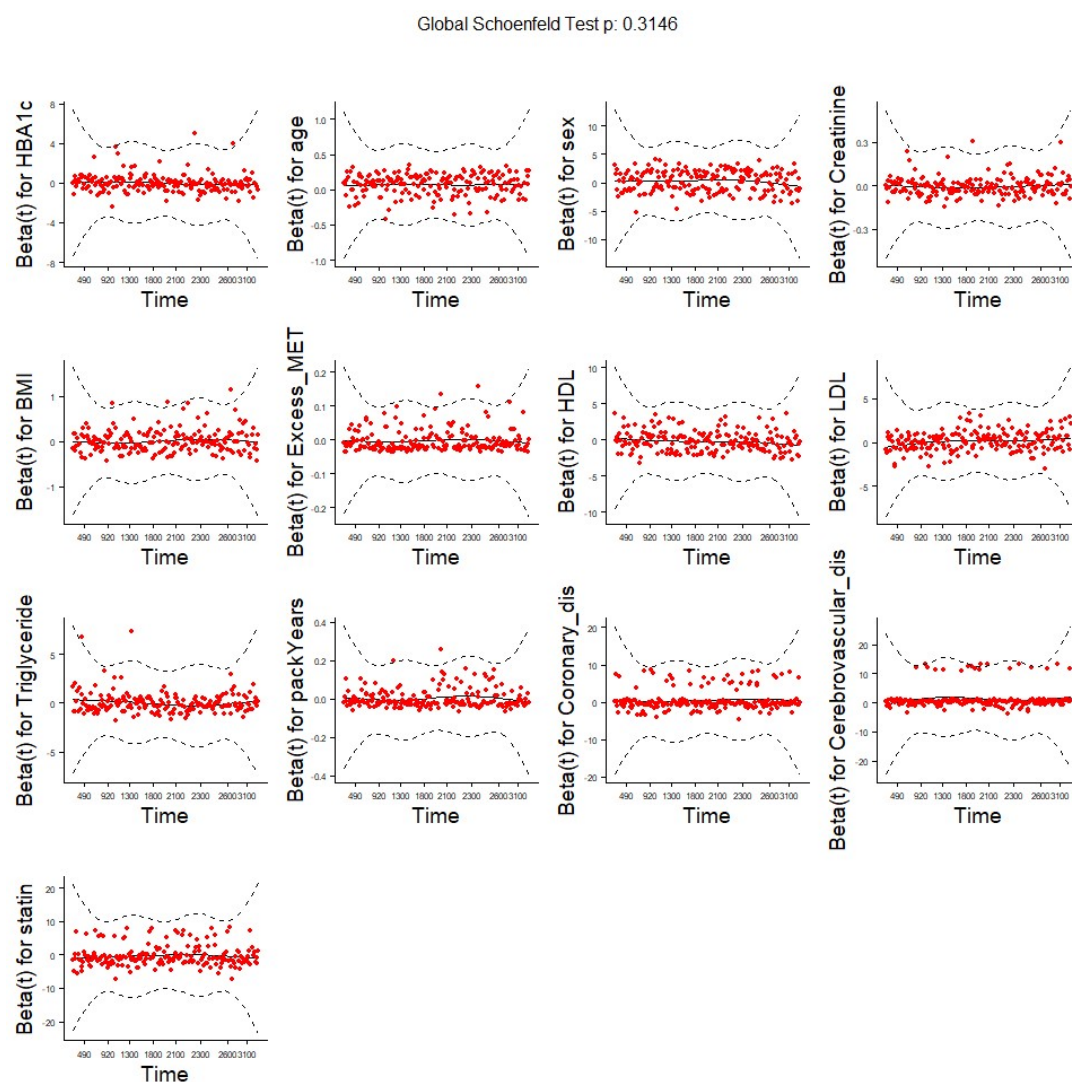

**Supplementary Figure 1** Schoenfeld residuals for variables included in combined model.

Supplement: Supplementary data [file jnnp-2021-327133supp001.pdf]
